# Trends in polypharmacy over 12 years and changes in its social gradients in South Korea

**DOI:** 10.1371/journal.pone.0204018

**Published:** 2018-09-18

**Authors:** Yeon-Hee Baek, Ju-Young Shin

**Affiliations:** School of Pharmacy, Sungkyunkwan University, Suwon, Gyeonggi-do, South Korea; La Trobe University, AUSTRALIA

## Abstract

Polypharmacy is associated with adverse drug reactions and represents an economic burden on the health insurance system. The objective of our study was to assess the trends in polypharmacy and its associated factors in South Korea. This cross-sectional study used a nationwide sampled database between 2002 and 2013, including outpatients of all ages who received at least 1 prescription in the same period. Polypharmacy was defined as the concomitant prescription of ≥6 distinct medications on a single prescription at least once without a given duration. The yearly prescribing trends were calculated and plotted. We conducted comparative analyses to identify the changes in social gradients of polypharmacy between the first 2 years, 2002‒2003, and the final 2 years, 2012‒2013. We repeated logistic regressions for pediatrics <20 years of age and adults ≥20 years of age to estimate the adjusted odds ratios (aOR) and 95% confidence intervals (CI). The distributions of polypharmacy in the respective periods were examined according to patient economic status (0 = most deprived and 10 = most affluent). The age-standardized prevalence of polypharmacy decreased from 65.8% in 2002 to 43.7% in 2013. Our study included 1,108,298 outpatients throughout 2002–2013. Pediatric patients aged 1–9 years had the highest number of medications among all age groups (mean: 5.1 ± 1.1 in 2002–2003 vs. 4.1 ± 1.1 in 2012–2013) in both periods. Changes in the association between deprivation and polypharmacy over 10 years were observed in adults (aOR = 0.68; 95% CI = 0.62–0.75 in 2002–2003 vs. 1.60; 95% CI = 1.54–1.66 in 2012–2013) and pediatrics (aOR = 0.60; 95% CI = 0.52–0.68 in 2002–2003 vs. 1.07; 95% CI = 1.01–1.14 in 2012–2013) compared with those in the most affluent patients. The high level of polypharmacy in pediatric patients is a public health concern that warrants policymaker attention.

## Introduction

Although appropriate polypharmacy based on the best evidence and optimized combinations of medicines may prolong a patient’s life expectancy [1], inappropriate polypharmacy has the potential to cause adverse clinical outcomes. Polypharmacy increases the risk of drug-drug interactions (DDIs), which in turn trigger adverse drug reactions (ADRs) [2]. Polypharmacy was observed to be associated with ADRs, including falls, mortality, or dementia, which have a devastating effect on patients’ quality of life [3–9]. Twenty-five percent of outpatients aged ≥18 years who received any prescription from participating primary care providers reportedly experienced an ADR, and their number of medications was significantly higher than that of the group without ADRs [10].

Multimorbidity is a well-known factor of polypharmacy and is largely associated with low socioeconomic status [11]. Patients with low socioeconomic status had an increased risk of mortality (risk ratio = 2.84; 95% confidence interval, CI = 2.25–3.60) [12], long working hours, and incident type 2 diabetes (risk ratio = 1.29; 95% CI = 1.06–1.57) [13]; a 2-fold increased risk of cardiovascular disease, coronary heart disease, and stroke [14]; and a 10- to 15-year earlier onset of multimorbidity [15]. Evaluating economic status is particularly important in polypharmacy research as it may affect multiple morbidity conditions and the consequent risk of polypharmacy [16].

Although most previous studies on polypharmacy focused on the elderly [8, 17, 18], evaluating polypharmacy in patients of all ages is essential for the following reasons. First, polypharmacy is largely driven by multimorbidity, and a significant number of people with multimorbidity are younger than 65 years, particularly in economically deprived populations [15]. Second, inappropriate medication overuse is highly prevalent in pediatric patients. Pediatrics younger than 9 years showed a more than 2-fold greater systematic antibiotic consumption than patients aged 10–59 years in South Korea [19]. Furthermore, 23% of pediatric ambulatory visits in the United States involved the inappropriate prescription of antibiotics for conditions that were unlikely to benefit from antibiotic treatment; this accounts for >10 million annual visits [20]. Thus, we included a population of all ages with the consideration of a potential earlier onset of multimorbidity in an economically deprived population, which in turn influences medication usage, and inappropriate medication overuse among pediatric patients.

Considering the increasing trends of polypharmacy in developed countries [21, 22], identifying the time-series trends of polypharmacy is an important public health issue, and policy efficacy must be evaluated to reduce polypharmacy. Therefore, the primary objective of our study was to present the longitudinal time trend of prescriptions in South Korea from 2002–2013 using a large sample obtained from the health care insurance database that represented the entire population. Our secondary objective was to identify and compare the associated social health gradients with polypharmacy over 10 years.

## Materials and methods

### Data source

We used the National Health Insurance Service-National Sample Cohort (NHIS-NSC), a population-based cohort established by the National Health Insurance Service (NHIS) in South Korea (Data number: NHIS-2018-2-024), and followed up from 2002–2013. After the initiation of the National Health Insurance program in Korea in 1977, it achieved universal coverage of the entire population in 1989. Accordingly, the database contains all information on health care use and prescribed drugs reimbursed by the NHIS for approximately 50 million Koreans. Information on non-reimbursed drugs or over-the-counter drugs is not covered by the database. The NHIS-NSC consists of approximately 1 million participants, corresponding to 2.2% of the entire Korean population, randomly selected from the NHI database [23]. The database includes demographic information on sex, level of income, and age groups, and clinical information on both inpatients and outpatients, drug prescriptions, and diagnoses of diseases coded based on the International Classification of Diseases, 10th Revision, Clinical Modification (ICD-10-CM).

### Study design and population

We carried out a cross-sectional study to estimate trends in polypharmacy over 12 years from 2002–2013. Comparison analyses between an earlier 2-year period (2002–2003) and the final 2-year period (2012–2013) were performed to compare the changes in the social determinants of polypharmacy. We included outpatients of all ages who received at least 1 prescription between 2002 and 2013 for the trend analysis. Patients with health care utilization records in the respective periods, either 2002–2003 or 2012–2013, were included in the comparative analyses.

We included outpatients (N = 1,108,298) between 2002 and 2013 to determine the annual polypharmacy trend. We respectively constructed cohorts of the first 2 years (2002–2003), in which we recruited 880,781 outpatients, and the final 2 years (2012–2013), corresponding to 953,648 outpatients. The number of pediatrics and adolescents (<20 years of age) was 258,559 in 2002–2003, and 206,668 in 2012–2013; that of adults and the elderly (≥20 years of age) was 622,222 and 746,980 in the respective periods.

### Outcomes

Our study included all prescriptions in outpatient settings that were reimbursed by the South Korean NHIS. Medications included both regular and pro re nata (prn) medications. Polypharmacy was defined as the prescription of ≥6 distinct medications concomitantly on a single prescription at least once in our study. This prescription-based definition of polypharmacy was used in order to align our study with a policy implemented to reduce overuse and misuse of prescribed medications in South Korea. We selected this definition without a given duration because acute respiratory tract infection was one of the reasons for polypharmacy in South Korea [24]. We classified the patients into polypharmacy (≥6 medications) and non-polypharmacy (≤5 medications) groups based on the maximum number of concomitantly prescribed medications. In the 12-year polypharmacy trend analyses between 2002 and 2013, the yearly prevalence of polypharmacy was calculated by dividing the sum of patients who received a polypharmacy prescription at least once by the total number of outpatients in the respective year. In the comparative analyses between the first 2 years (2002–2003) and the final 2 years (2012–2013), patients who received a polypharmacy prescription at least once during the respective period were defined to have an outcome.

### Covariates

Multimorbidity was defined as the coexistence of 2 or more morbidities [15]. The definition of multimorbidity included the 51 most frequently reported chronic conditions by health care systems in Germany [25] and South Korea [26]. We used an unweighted count of morbidity as no single outcome was specifically selected for our purpose to describe the general morbidity status in the population. The conditions included physical comorbidities across system organ classes, including hypertension, diabetes, osteoporosis, chronic gastritis, liver disease, thyroid dysfunction, and osteoporosis, in addition to mental disorders, including dementia, anxiety, and depression (S1 File).

In addition to multimorbidity, we also measured comorbidities and the Charlson Comorbidity Index (CCI). We selected highly prevalent chronic conditions to compare the comorbidity status between the polypharmacy and non-polypharmacy groups; the comorbidities included hypertension (ICD-10: I10–I15), diabetes (ICD-10: E10–E14), hyperlipidemia (ICD-10: E78), ischemic heart disease (ICD-10: I20–I25), cerebrovascular disease (ICD-10: I60–I68), arterial disease (ICD-10: I70–I79), and kidney disease (ICD-10: N02–N05, N18, N19, N25, N391, I132, I139, I150, and I151). Contrary to multimorbidity, which involved unweighted counts of morbidity, we measured the severity of morbidity using the CCI [27], a weighted predictor of mortality.

We used the latest records for individual patients in the first 2 years (2002–2003) and the final 2 years (2012–2013) to measure their age and level of income for the analyses. Age groups were divided into 10-year intervals ranging from 0–85 years or older to demonstrate the representative polypharmacy status of each generation in the graphs showing the distribution of polypharmacy and the logistic regression analysis.

Economic status was defined by the insurance fee, which is priced according to the level of income and properties, as assessed by the Korean NHIS. It consists of 11 deciles, ranging from 0 (the most deprived) to 10 (the most affluent). The 0th decile corresponds to medical aid patients and the most deprived population. The deciles are divided by 10% intervals ranging from the first to the tenth deciles in order, in which the first decile accounts for the lowest 10% and the tenth decile the highest 10%.

### Statistical analysis

The prescription trends over 12 years between 2002 and 2013 were plotted and presented graphically. To assess the 12-year polypharmacy trends between 2002 and 2013, the yearly prevalence of polypharmacy was calculated by dividing the sum of patients who received a polypharmacy prescription at least once by the total number of outpatients in the respective year. As the distributions of age were heterogeneous across the years, the age-standardized prevalence of polypharmacy was calculated using the values of 2013, the last year of our study, as a reference. We calculated the prevalence of polypharmacy in each 10-year age interval of the standard population (2013). The expected number of patients exposed to polypharmacy in each year from 2002–2013 was respectively calculated across the age groups. We computed the standardized polypharmacy ratio using the sum of actual polypharmacy patients in each year as the numerator and the sum of expected polypharmacy patients as the denominator. The age-standardized prevalence of polypharmacy was calculated by multiplying the yearly prevalence by the calculated ratios.

The number of prescriptions and the mean number of medications were calculated on a yearly basis. The yearly changes in the mean number of medications per prescription and the age-standardized prevalence were plotted graphically. To examine the distributions in polypharmacy by age and income groups in the respective periods, we respectively plotted the association between polypharmacy and age in the first 2 years (2002–2003) and the final 2 years (2012–2013); the x-axis corresponded to the age in 10-year intervals and the y-axis is the mean number of medications. The plots were presented according to the economic status scale (0 = most deprived and 10 = most affluent). We calculated the mean medication use for each patient.

Patient characteristics, including age, sex, level of income, multimorbidity, CCI, and comorbidities, were summarized as counts with proportions for categorical variables and means with standard deviations (SD) for continuous variables between the polypharmacy and non-polypharmacy groups. Chi-square tests were used to test for significant differences between groups.

We conducted a logistic regression analysis to calculate the odds ratios (aOR) and 95% CIs of the associations between patient characteristics and polypharmacy. We repeated the logistic regression analyses for pediatrics and adolescents (<20 years of age) and adults and the elderly (≥20 years of age) in the first 2 years (2002–2003) and the final 2 years (2012–2013). Models were adjusted for sex, age group, level of income, and multimorbidity. To avoid multicollinearity, we excluded the following variables from the logistic model: comorbidity and CCI, which overlapped with multimorbidity, and health insurance type, a surrogate variable for the level of income. Aside from multimorbidity, comorbidity comprised of 7 highly prevalent morbidities: hypertension, diabetes, hyperlipidemia, ischemic heart disease, cerebrovascular disease, arterial disease, and kidney disease. We carried out a likelihood ratio test for the estimation of the p-for-trend between economic status and multimorbidity. All statistical procedures were performed using SAS version 9.4 (SAS Institute Inc., Cary, NC, USA).

### Sensitivity analysis

We applied a threshold for polypharmacy of as ≥5 simultaneously prescribed medications in a single prescription to evaluate the robustness of the results of polypharmacy trends. To assess the weight of cancer patients contributing to the prevalence of polypharmacy, we limited study subjects to non-cancer patients in the sensitivity analysis. Patients with diagnostic records of cancer (ICD-10: C00–C97) in the outpatient setting within the respective year were excluded from the analysis. The polypharmacy trends in non-cancer patients were presented with the yearly age-standardized prevalence and the respective prevalence in pediatrics and adolescents (<20 years of age) and adults and the elderly (≥20 years of age) across 2002–2013.

As the results may have been influenced by two extreme categories (most deprived compared to most affluent groups on a 10-item scale), we conducted a sensitivity analysis with patients’ insurance type in the NHIS, either medical aid recipients (0th decile) or National Health Insurance beneficiaries (1st decile-10th decile), instead of using income deciles. In this analysis, the insurance type, either medical aid recipient or National Health Insurance beneficiary, was binary coded, and the logistic model was adjusted for sex, insurance type, and multimorbidity.

## Results

**Fig 1:**
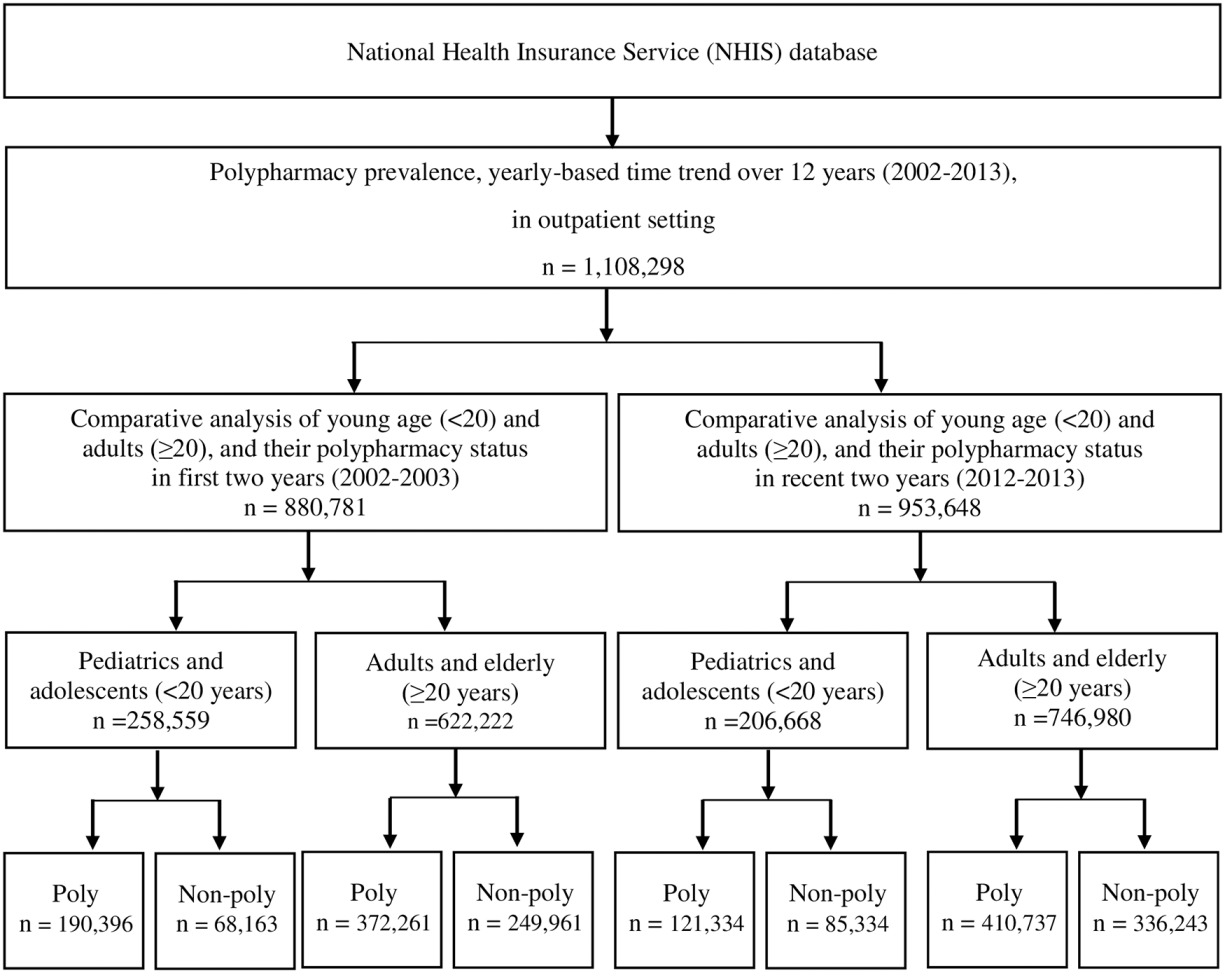
Study flowchart for 12-year polypharmacy trends and the comparative analysis of polypharmacy and non-polypharmacy between 2002–2003 and 2012–2013. Polypharmacy was defined as ≥6 medications and non-polypharmacy as ≤5 medications, based on the maximum number of concomitantly prescribed medications.

**Fig 2:**
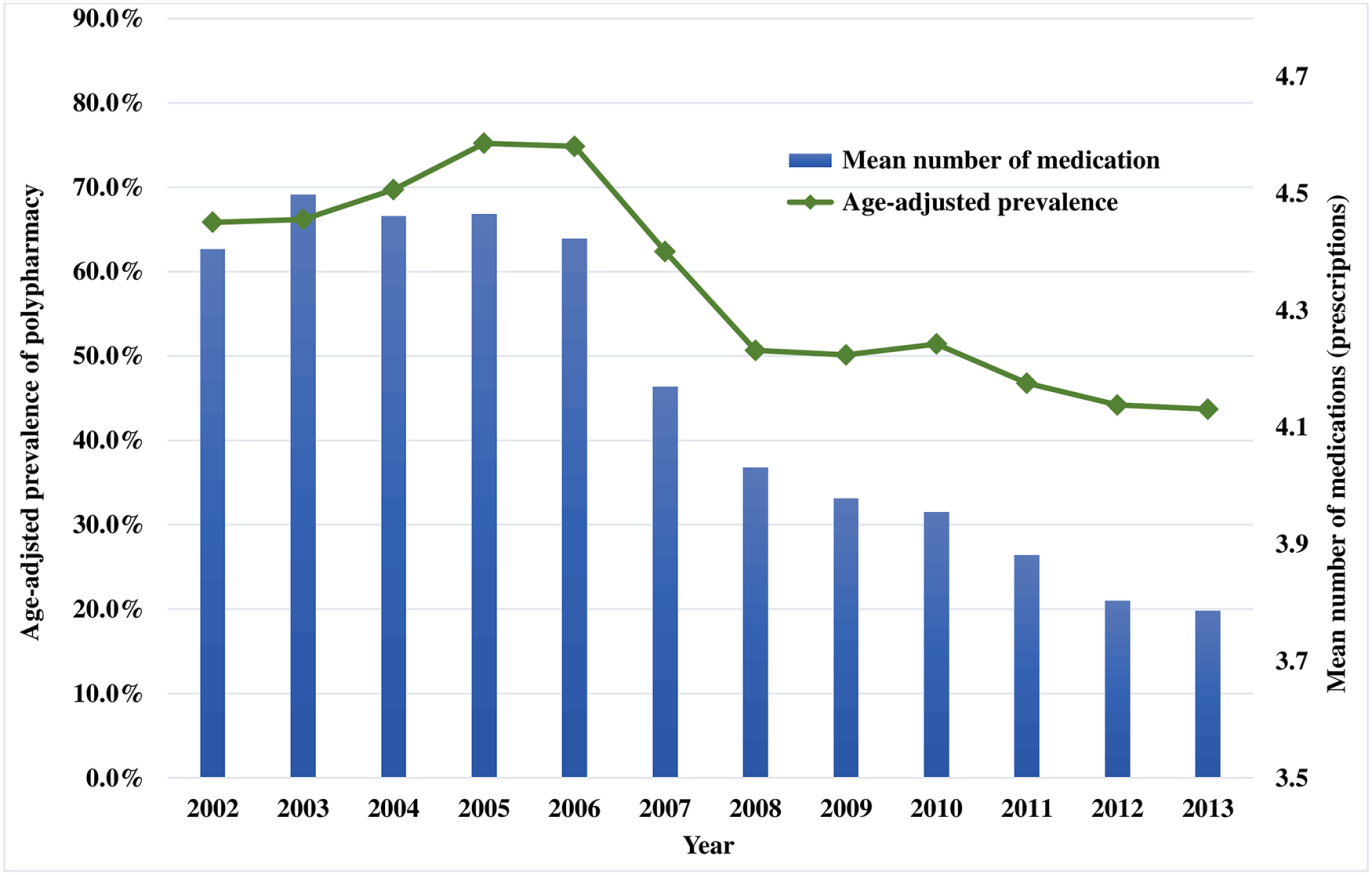
Changes in age-adjusted prevalence of polypharmacy and the mean number of medications per prescription between 2002 and 2013 in Korea.

Our study observed decreasing trends in polypharmacy for 1,108,298 outpatients (Fig 1). The prevalence of polypharmacy and the mean number of medications showed a decreasing trend (Fig 2). The age-standardized prevalence decreased from 65.8% in 2002 to 43.7% in 2013; that of pediatrics and adolescents (<20 years of age) decreased from 65.3% to 46.6% and that of adults and the elderly (≥20 years of age) decreased from 48.4% to 42.9% (Table 1). The mean number of medications in prescriptions decreased from 4.4 (SD = 2.0) in 2002 to 3.8 (SD = 1.8) in 2013. The number of prescriptions steadily increased over the 12-year period; the number doubled from 4.57 million in 2002 to 9.98 million in 2013 (S2 File), and the average number of prescriptions over the period was 7.34 million. The decreasing trends in polypharmacy was consistent in the sensitivity analyses performed with a polypharmacy threshold of ≥5 medications in a single prescription (S3 File) and restricting the population to non-cancer patients (S4 File).

**Table 1:** Changes in the prevalence of polypharmacy in pediatrics and adolescents (<20 years of age) and in adults and the elderly (≥20 years of age) between 2002 and 2013.

	Age-standardized prevalence*	Pediatrics and adolescents (<20 years)			Adults and elderly (≥20 years)		
		Prevalence^†^	Total (N)	Poly (N) ^‡^	Prevalence^†^	Total (N)	Poly (N) ^‡^
**Year**							
2002	65.8%	65.3%	236,399	154,268	48.4%	533,648	258,290
2003	66.2%	62.7%	219,141	137,355	50.2%	543,067	272,370
2004	69.7%	64.0%	219,939	140,697	51.5%	565,998	291,726
2005	75.2%	64.9%	216,886	140,738	54.1%	588,092	318,332
2006	74.8%	64.4%	210,687	135,748	54.1%	591,303	319,724
2007	62.4%	56.2%	208,884	117,428	50.3%	614,901	309,055
2008	50.6%	50.2%	204,662	102,802	45.6%	624,777	284,871
2009	50.1%	52.2%	208,518	108,797	44.6%	639,253	285,131
2010	51.4%	53.0%	199,900	105,919	45.1%	635,737	286,982
2011	46.8%	48.4%	200,928	97,218	43.9%	667,742	293,418
2012	44.2%	46.8%	197,825	92,570	43.0%	680,235	292,161
2013	43.7%	46.6%	192,172	89,642	42.9%	687,607	294,695

Abbreviations: Poly, polypharmacy.

* Yearly prevalence was standardized to the age distributions in 2013.

^†^ Prevalence was calculated by dividing the sum of polypharmacy patients by the total number of outpatients in the respective year.

^**‡**^ Polypharmacy was defined as the concomitant prescription of ≥6 distinct medications on a single prescription without a given duration of time.

We described the demographic characteristics of the study populations in the first 2 years (2002–2003) and the final 2 years (2012–2013) according to their polypharmacy status (Table 2). The proportion of patients with polypharmacy was higher in 2002–2003 than in 2012–2013 (63.9% vs. 55.8%). Compared with non-polypharmacy, higher proportions of polypharmacy were observed in women (55.5% in 2002–2003, 55% in 2012–2013), pediatric patients aged 1–9 years (19.4% vs. 4.5% in 2002–2003, 12.3% vs. 3.3% in 2012–2013), elderly patients aged ≥60 years (13.6% vs. 8.9% in 2002–2003, 23.2% vs. 12.2% in 2012–2013), those with multimorbidity (65.1% vs. 30.0% in 2002–2003, 88.5% vs. 60.6% in 2012–2013), and in all comorbidities (Table 2). In contrast, in the most deprived group of patients, the rate of polypharmacy was lower than that of non-polypharmacy in 2002–2003, while the opposite results were shown in 2012–2013 (0.2% vs. 0.5% in 2002–2003, 3.8% vs. 2.1% in 2012–2013).

**Table 2:** Baseline characteristics of outpatients in the Korea National Health Insurance Service–National Sample Cohort according to medication use in the first years of the study, 2002–2003, and the recent years of the study, 2012–2013.

	2002–2003 (N = 880,781)		2012–2013 (N = 953,648)		P-value
	Poly^†^ (n = 562,657)	Non-poly (n = 318,124)	Poly^†^ (n = 532,071)	Non-poly (n = 421,577)	
	n (%)	n (%)	n (%)	n (%)	
**Sex**					
Male	250,534 (44.5)	170,701 (53.7)	239,476 (45.0)	225,648 (53.5)	<0.001
Female	312,123 (55.5)	147,423 (46.3)	292,595 (55.0)	195,929 (46.5)	
**Age group (years)**					
0	12,397 (2.2)	3,280 (1.0)	2,155 (0.4)	5,088 (1.2)	<0.001
1–9	108,890 (19.4)	14,361 (4.5)	65,466 (12.3)	13,869 (3.3)	
10–19	69,109 (12.3)	50,522 (15.9)	53,713 (10.1)	66,377 (15.7)	
20–29	64,817 (11.5)	67,639 (21.3)	51,146 (9.6)	67,083 (15.9)	
30–39	91,169 (16.2)	67,068 (21.1)	70,799 (13.3)	71,779 (17.0)	
40–49	83,801 (14.9)	57,430 (18.1)	80,015 (15.0)	81,371 (19.3)	
50–59	55,748 (9.9)	29,384 (9.2)	85,367 (16.0)	64,391 (15.3)	
60–69	48,832 (8.7)	18,194 (5.7)	58,541 (11.0)	28,390 (6.7)	
70–79	22,072 (3.9)	7,206 (2.3)	46,839 (8.8)	15,858 (3.8)	
≥80	5,822 (1.0)	3,040 (1.0)	18,030 (3.4)	7,371 (1.7)	
**Health insurance type**					
Medical aid	1,339 (0.2)	1,640 (0.5)	20,046 (3.8)	8,909 (2.1)	<0.001
NHI beneficiary	561,318 (99.8)	316,484 (99.5)	512,025 (96.2)	412,668 (97.9)	
**Level of income**					
0 (Medical aid, most deprived)	1,339 (0.2)	1,640 (0.5)	20,046 (3.8)	8,909 (2.1)	<0.001
1	32,659 (5.8)	19,547 (6.1)	37,214 (7.0)	27,159 (6.4)	
2	31,682 (5.6)	20,808 (6.5)	35,798 (6.7)	28,386 (6.7)	
3	38,147 (6.8)	24,056 (7.6)	35,723 (6.7)	30,439 (7.2)	
4	45,159 (8.0)	27,446 (8.6)	38,749 (7.3)	33,067 (7.8)	
5	53,109 (9.4)	30,217 (9.5)	43,152 (8.1)	36,285 (8.6)	
6	61,037 (10.8)	32,971 (10.4)	49,663 (9.3)	40,501 (9.6)	
7	68,089 (12.1)	35,757 (11.2)	56,251 (10.6)	44,547 (10.6)	
8	76,161 (13.5)	38,219 (12.0)	65,324 (12.3)	49,332 (11.7)	
9	78,739 (14.0)	42,145 (13.2)	73,724 (13.9)	57,896 (13.7)	
10 (most affluent)	76,536 (13.6)	45,318 (14.2)	76,427 (14.4)	65,056 (15.4)	
**Multimorbidity**					
0	66,881 (11.9)	107,306 (33.7)	11,997 (2.3)	55,174 (13.1)	<0.001
1	129,742 (23.1)	115,465 (36.3)	49,124 (9.2)	111,108 (26.4)	
2–4	274,549 (48.8)	87,403 (27.5)	262,988 (49.4)	204,039 (48.4)	
5–7	69,722 (12.4)	7,276 (2.3)	127,945 (24.0)	42,476 (10.1)	
≥8	21,763 (3.9)	674 (0.2)	80,017 (15.0)	8,780 (2.1)	
**CCI (n, %)**					
0	316,130 (56.2)	264,577 (83.2)	235,973 (44.3)	312,407 (74.1)	<0.001
1–2	223,056 (39.6)	50,394 (15.8)	247,193 (46.5)	100,249 (23.8)	
3–5	21,660 (3.8)	2,701 (0.8)	44,345 (8.3)	8,183 (1.9)	
>5	1,811 (0.3)	452 (0.1)	4,560 (0.9)	738 (0.2)	
**Comorbidities**					
Hypertension	63,651 (11.3)	16,030 (5.0)	112,546 (21.2)	38,492 (9.1)	<0.001
Diabetes	28,209 (5.0)	7,048 (2.2)	51,453 (9.7)	14,393 (3.4)	
Hyperlipidemia	18,518 (3.3)	4,890 (1.5)	67,081 (12.6)	24,087 (5.7)	
Ischemic heart disease	14,679 (2.6)	2,958 (0.9)	21,946 (4.1)	4,838 (1.1)	
Cerebrovascular disease	7,812 (1.4)	1,813 (0.6)	18,037 (3.4)	4,814 (1.1)	
Arteries disease	8,594 (1.5)	1,455 (0.5)	26,891 (5.1)	7,162 (1.7)	
Kidney disease	3,856 (0.7)	1,090 (0.3)	5,084 (1.0)	1,947 (0.5)	

Abbreviations: Poly, polypharmacy; non-poly, non-polypharmacy; NHI, National Health Insurance; CCI, Charlson comorbidity index.

^**†**^ Polypharmacy was defined as the concomitant prescription of ≥6 distinct medications on a single prescription without a given duration of time.

**Fig 3:**
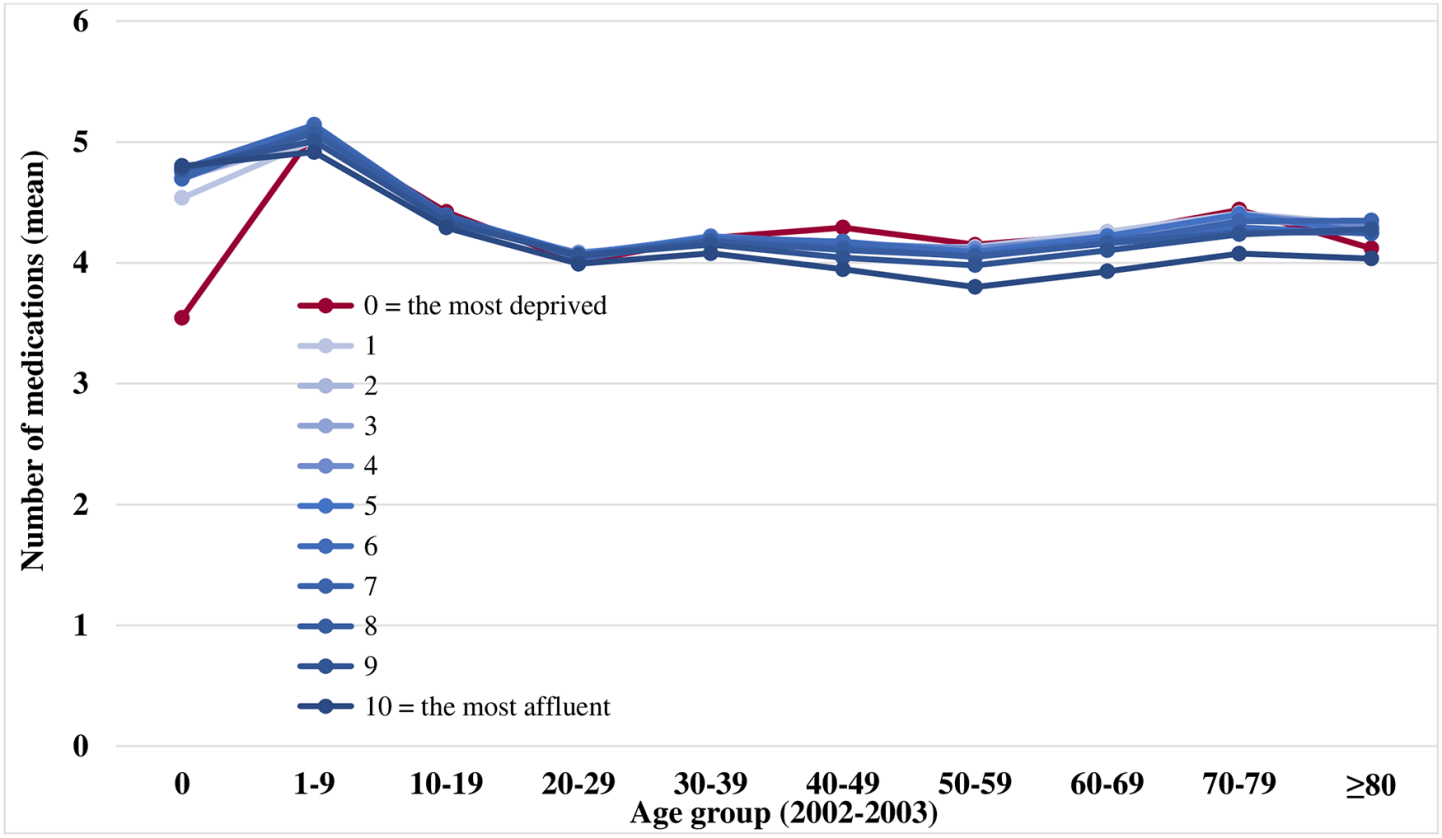
Distributions of medication use (mean) by age and economic status, 2002–2003. Economic status scale: 0 = most deprived and 10 = most affluent.

**Fig 4:**
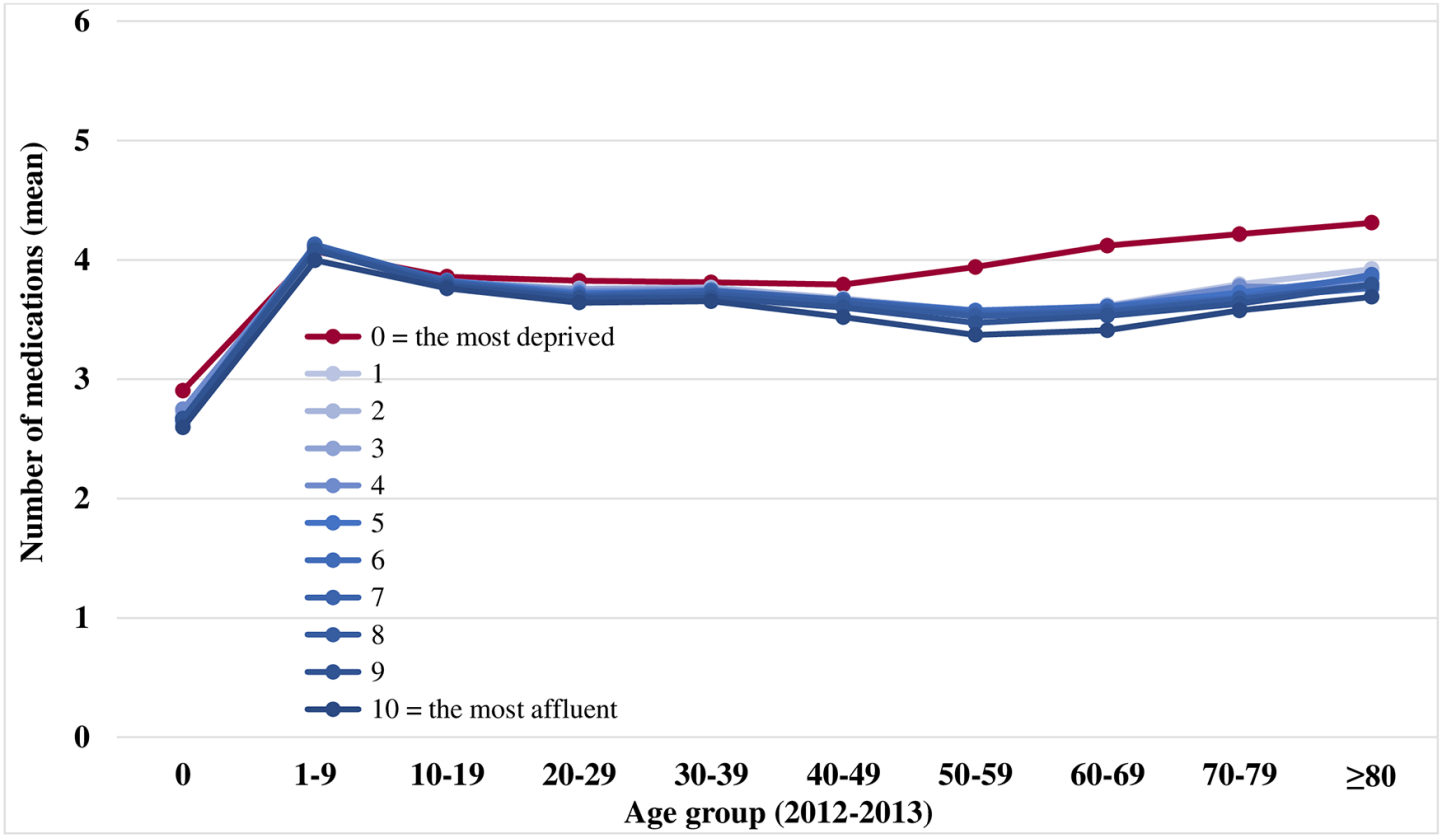
Distributions of medication use (mean) by age and economic status, 2012–2013. Economic status scale: 0 = most deprived and 10 = most affluent.

Figs 3 and 4 show the distributions of medication usage (mean) according to age group and income level. Pediatric patients aged 1–9 years had the highest mean number of medications among all age groups (5.1 ± 1.1 in 2002–2003 vs. 4.1 ± 0.8 in 2012–2013). The most deprived participants did not have higher medication consumption than other deciles across all age groups in 2002–2003 (Fig 3); moreover, infants aged 0 years had a significantly lower mean number of medications. However, the most deprived patients had a higher mean number of medications than other deciles in 2012–2013 (Fig 4).

In the logistic regression analysis of polypharmacy status (Table 3), we observed discrepant findings in the association between the level of income and polypharmacy in the 2 study periods. In 2002–2003, the most deprived participants had a decreased risk of polypharmacy compared with the most affluent participants in both pediatrics and adolescents (aOR = 0.60; 95% CI = 0.52–0.68) and adults and the elderly (aOR = 0.68; 95% CI = 0.62–0.75). Conversely, we observed a non-significant risk in pediatrics and adolescents (aOR = 1.07; 95% CI = 1.01–1.14) and an increased risk in adults and the elderly (aOR = 1.60; 95% CI = 1.54–1.66) in 2012–2013. The odds of polypharmacy generally increased with multimorbidity across study periods and age groups (Table 3). The trends between economic status and multimorbidity were statistically significantly different with a P-value of <0.05 (results not shown). The directions of the association were consistent in the sensitivity analysis using insurance type, binary coded for either medical aid recipients (0th decile) or National Health Insurance beneficiaries (1st decile-10th decile) (S5 File).

**Table 3:** Logistic regression model of the association between participant characteristics and polypharmacy (≥6 medications) in 2002–2003 and 2012–2013.

	2002–2003 (N = 880,781)		2012–2013 (N = 953,648)	
	Pediatrics and adolescents (<20) N = 258,559 aOR (95% CI)	Adults and elderly (≥20) N = 622,222 aOR (95% CI)	Pediatrics and adolescents (<20) N = 206,668 aOR (95% CI)	Adults and elderly (≥20) N = 746,980 aOR (95% CI)
**Male (reference)**				
Female	1.09 (1.07–1.11)	1.38 (1.37–1.40)	1.03 (1.01–1.05)	1.24 (1.23–1.25)
**Level of income**^†^				
**10 (reference)**				
0 (Medical aid, most deprived)	0.60 (0.52–0.68)	0.68 (0.62–0.75)	1.07 (1.01–1.14)	1.60 (1.54–1.66)
1	0.92 (0.88–0.97)	1.12 (1.09–1.15)	1.06 (1.02–1.11)	1.17 (1.14–1.20)
2	0.99 (0.94–1.04)	1.09 (1.06–1.12)	1.07 (1.03–1.13)	1.19 (1.16–1.22)
3	1.10 (1.05–1.15)	1.09 (1.06–1.12)	1.01 (0.96–1.05)	1.16 (1.13–1.18)
4	1.24 (1.19–1.30)	1.12 (1.09–1.14)	1.11 (1.06–1.16)	1.16 (1.14–1.19)
5	1.34 (1.29–1.40)	1.15 (1.13–1.18)	1.22 (1.17–1.27)	1.17 (1.14–1.19)
6	1.40 (1.35–1.45)	1.17 (1.15–1.20)	1.26 (1.21–1.31)	1.18 (1.15–1.20)
7	1.41 (1.36–1.46)	1.16 (1.14–1.19)	1.38 (1.33–1.43)	1.17 (1.14–1.19)
8	1.42 (1.37–1.47)	1.19 (1.16–1.22)	1.40 (1.35–1.45)	1.17 (1.14–1.19)
9	1.19 (1.15–1.23)	1.12 (1.10–1.15)	1.15 (1.12–1.19)	1.12 (1.10–1.14)
**Multimorbidity**				
**0 (reference)**				
1	2.05 (2.01–2.09)	1.77 (1.74–1.80)	2.39 (2.30–2.49)	1.87 (1.82–1.92)
2–4	7.43 (7.25–7.62)	5.25 (5.17–5.34)	7.98 (7.70–8.28)	5.34 (5.20–5.47)
5–7	23.62 (19.35–28.83)	20.26 (19.71–20.83)	15.95 (14.96–17.00)	14.78 (14.39–15.18)
≥8	7.22 (2.17–24.10)	68.65 (63.50–74.21)	27.45 (17.38–43.35)	44.63 (43.18–46.12)

Abbreviations: aOR, adjusted odds ratio; CI, confidence interval.

^†^ Level of income was classified by the economic status scale: 0 = most deprived and 10 = most affluent.

## Discussion

Our study was designed to present long-term polypharmacy trends, their distributions, and changes in associating factors over 10 years. This real-world evidence showed gradually decreasing trends in polypharmacy over 12 years. Pediatric patients aged 1–9 years had the highest mean number of medications in both the first 2 years (2002–2003) and the final 2 years (2012–2013), highlighting the importance of medication guidelines to reduce the number of medications prescribed for pediatric patients. In addition, inconsistencies in the association between economic deprivation and polypharmacy were observed between the 2 periods; inverse associations were shown in both pediatrics <20 years of age and adults ≥20 years of age in 2002–2003. Conversely, a 1.6-fold increased risk in adults and an insignificant association in pediatrics were observed in 2012–2013.

We observed a decreasing trend in the age-standardized prevalence of polypharmacy, from 65.8% in 2002 to 43.7% in 2013. The decreasing trend was consistent in pediatrics from 65.3% in 2002 to 46.6% in 2013 and adults from 48.4% to 42.9%. The result was decreased prescription medication use among patients of all age groups in Korea, from 4.4 ± 2.0 in 2002 to 3.8 ± 1.8 in 2013. This decrease reflects efforts to reduce prescriptions made by the Korean Public Health Authority, which adopted the Evaluation Project on Appropriate Prescribing (EPAP) in 2001 to reduce the misuse and abuse of prescription medicines by improving the autonomous management of health institutions [28]. Apart from the decreasing trends in polypharmacy, the observed increase in the number of prescriptions may be the result of the aging of the study cohort.

Our finding of a decreasing trend in polypharmacy is contrary to that of other studies that used a single prescription-based definition and reported an increase in polypharmacy over time. A study in the United States reported that the prevalence of polypharmacy (≥5 medications) rose from 8.2% to 15% overall between 1999 and 2012 and from 24% to 39% in participants aged ≥65 years [29]. An increase was also observed in Scotland among those ≥20 years of age, from 11.4% to 20.8% between 1995 and 2010 (≥5 medications) [30], and from 24.7% to 31.6% in New Zealand between 2005 and 2013 among elderly people aged ≥65 years [31]. This conflict in trends may be due to the high level of polypharmacy in the Korean population. South Korea had more drug items per prescription (mean: 4.16) than the United States (mean: 1.97), United Kingdom (mean: 3.83), Japan (mean: 3.00), France (mean: 4.02), and Germany (mean: 1.98) in 2005 [24]. One reason for the very high number of drugs on a single prescription in Korea may be the high use of medications for acute upper respiratory infection (mean: 4.73) [24]. Although a decreasing trend in polypharmacy was observed in South Korea, the magnitude of the prevalence remained high.

Of note is that the pediatric patients 1–9 years of age had the highest number of medications among all age groups. This is well-supported by a finding that children ≤6 years of age had a higher level of antibiotic consumption than adults in South Korea [32], while the reverse was found in other Organization for Economic Co-operation and Development (OECD) countries [24]. Moreover, infants in Korea had the highest antibiotic use among 6 countries (Germany, Italy, South Korea, Norway, Spain, and the United States) [33]. The medications most frequently associated with pediatric patients were treatments for the common cold throughout the first years of our study, 2002–2003, and the recent years, 2012–2013 (S6 File). Although our study observed a decreasing trend in polypharmacy in pediatrics <20 years of age from 65.3% in 2002 to 46.6% in 2013, antibiotic consumption was still prevalent in this population in the last years of our study. Our results suggest the need for subsequent studies focusing on medication use in pediatrics and prescription guidelines in clinical practice to reduce polypharmacy in the population.

The highest medication consumption in pediatric patients is discordant with the positive association between the number of prescription medications and increasing age observed in the United States [34], where pediatrics aged ≤18 years had the lowest proportion of ≤5 prescription drugs among all age groups, and in Canada [35], where the proportion of ≤5 prescription drugs increased with age. Polypharmacy in pediatric patients could be particularly problematic considering their physical vulnerabilities [36, 37] and higher risk of potential DDIs owing to the differences in their response to drugs compared with adults [38]. Pediatrics can react differently to drug administration as their pharmacokinetic processes of absorption, distribution, metabolism, and excretion are different from those of adults [39].

Contrary to our hypothesis that deprivation accelerates multimorbidity and its consequent polypharmacy, our results showed associations between deprivation and polypharmacy independently from multimorbidity. Adults in the most deprived group had a 40% decreased risk of polypharmacy compared with the most affluent in 2002–2003, whereas the most deprived had a 60% increased risk of polypharmacy in 2012–2013. This reversed association over 10 years may be attributed to the decrease in medical utilization among the reference group, the most affluent, due to differences in the cost-sharing policy in South Korea between the study periods. This may have influenced the relatively higher medical utilization among the most deprived patients, and consequently increased the odds of polypharmacy after the policy reform. The cost-sharing policy was reformed in 2007 from copayment to coinsurance for National Health Insurance beneficiaries younger than 65 years, with the exclusion of medical aid patients corresponding to the 0th decile, i.e., those who are the most deprived. In order to reduce the burden of rapidly increasing health expenditures after the initiation of the National Health Insurance program in 1989 [40], a patient cost-sharing change was implemented for outpatients aged younger than 65 years on August 1, 2007. In the copayment system, outpatients had a fixed medical expense of KRW (Korean won) 3,000 from their own money unless the total costs exceeded the threshold of KRW 15,000; if the total costs were greater than the threshold, patients were obligated to pay 30% [41]. After the coinsurance policy was implemented, national health beneficiaries (1st decile–10th decile) shared 30% of the total costs from their own money, regardless of total costs [42], whereas the copayment system was still applied to medical aid patients. The NHIS estimated that the average out-of-pocket cost for primary care visits for the common cold increased from KRW 3,110 to KRW 3,300 after the reform and prospected that the burden on the NHIS fund would be reduced by USD 193 million, annually [43].

In 2012–2013, after the change to the coinsurance system, decreased health care utilization among the most affluent patients may have influenced the relatively higher health care utilization in the most deprived group, resulting in an increased OR. Our interpretation is in line with a previous finding that medical care utilization, defined as the proportion of all beneficiaries in each group who visited clinics and the mean number of visit days per beneficiary, decreased after the cost-sharing change to coinsurance in South Korea [41]. Moreover, medical accessibility for the most deprived participants was enhanced by the coverage expansion of the Medical Aid Program to patients with rare, intractable, and chronic diseases, as well as children under the age of 18 in 2004 [44]. The increased association between medical aid recipients and polypharmacy in 2012–2013 also corresponds with a South Korean study that estimated a 52% increased risk of polypharmacy among elderly outpatients receiving medical aid in 2010–2011 [45], although the age inclusion criterion was different. Our study has several strengths. We generated generalizable results by using a representative electronic health insurance database. In addition, we determined the trends in polypharmacy between 2002 and 2013 using longitudinal data over a 12-year period. This is particularly important evidence to evaluate the outcome of South Korea initiating the EPAP in 2001 [28] to reduce the misuse or overuse of prescription medications. To the best of our knowledge, this is the first study presenting long-term trends for polypharmacy in a population of all ages in South Korea. By evaluating polypharmacy in a population of all ages, we identified that pediatric patients aged 1–9 years had the highest medication use. The economic status used in our study was objective and trustworthy as it was assessed by the Korean Public Health Authority according to the level of income and private properties, including real estate and vehicles. The monthly contribution is calculated by the monthly average wage multiplied by contribution rate for employee health insurance subscribers, and by the contribution score multiplied by the unit price for those with self-employed health insurance [46].

However, our study should be interpreted with caution owing to the following limitations. The definition of polypharmacy was based on the maximum number of concomitant medications on a prescription without a given duration. The prescription-based definition of polypharmacy might lead to an underestimation of polypharmacy by failing to capture doctor-shopping behavior. For example, patients who were simultaneously prescribed with <6 medications in multiple primary care institutions will be classified in the non-polypharmacy group even if the sum of prescribed medications for the different institutions exceeded ≥6, the threshold of polypharmacy. This definition may also lead to an overestimation of polypharmacy by the assessment without a given duration of time. For example, patients with short-term polypharmacy prescriptions will be classified in the polypharmacy group. A definition reflecting the duration of prescriptions may have been more appropriate for the medications prescribed for chronic disease. It is particularly problematic when comparing the results with studies using a duration-based definition. Discrepancies in the definition of polypharmacy were observed; for example, some studies defined polypharmacy as at least 5 medications [47, 48], 6 medications [49, 50], 10 medications [51], or used operational definitions, such as polypharmacy, that continued for more than a given duration of time [52–54].

However, for our objective to capture all prescriptions in primary care, we judged this prescription-based definition of polypharmacy to be more appropriate. Hence, the definition enabled our study to capture the medications prescribed for a short duration that are largely overused in the outpatient setting in South Korea, such as antibiotics [55], analgesics, and nonsteroidal anti-inflammatory drugs [56]. Our polypharmacy definition was aligned with the EPAP indicator in South Korea, a policy to reduce the misuse and abuse of prescription medicines. For polypharmacy, the EPAP indicator was defined as ≥6 drugs prescribed concomitantly on 1 prescription [28, 57]. To evaluate longitudinal polypharmacy trends and the efficacy of policies, we aligned our polypharmacy definition with the policy. Moreover, the majority of former polypharmacy studies used a prescription-based definition without a given duration of time [58]. According to a systematic review exploring the definitions of polypharmacy, only 9 studies considered a given duration of time among 99 included studies [58]. Thus, we judged the prescription-based definition of polypharmacy to be more appropriate to increase comparability with previous findings.

The limitation of excluding non-reimbursed medications or over-the-counter drugs may lead to an underestimation of polypharmacy. However, this is not a significant hurdle as our study’s objective was to measure polypharmacy of drugs reimbursed by the health care system. Furthermore, problems in interpreting the results may occur by combining multilevel variables in the multivariate logistic regression analyses. Our outcome, polypharmacy, involves multiple stakeholders, including individual patients, medical institutions, and health care systems, which is contrary to individual-level independent variables, such as age, sex, income, and multimorbidity. Other important unmeasured factors that may have an association with polypharmacy, such as alcohol consumption [59] or smoking status [21], were not controlled for in the analyses. Finally, the limitation of using prescription data underlies our study, as we do not know if patients actually took the medications prescribed.

In conclusion, our study observed that the prevalence of polypharmacy has declined over 12 years in South Korea. We provided evidence demonstrating that the highest risk of polypharmacy across all age groups was in pediatric patients 1–9 years of age. Considering the risk of polypharmacy and the vulnerability of pediatric patients, high medication use in this population is an important public health concern that should warrant the attention of policymakers and clinicians, as well as further research. Implementation of strategies to manage polypharmacy will be critical for safe and quality care in this population.

## Supporting information

S1 File The 51 chronic conditions included in the multimorbidity count. (DOCX)

S2 File Changes in the prescription trends of polypharmacy and non-polypharmacy between 2002 and 2013. (DOCX)

S3 File Changes in the prevalence of polypharmacy, defined as ≥5 medications, in pediatrics and adolescents (<20 years of age) and in adults and the elderly (≥20 years of age) between 2002 and 2013. (DOCX)

S4 File Changes in the prevalence of polypharmacy in non-cancer patients between 2002 and 2013. (DOCX)

S5 File Sensitivity analysis for the association between participant characteristics, including insurance type, and polypharmacy (≥6 medications) in 2002–2003 and 2012–2013. (DOCX)

S6 File The 20 most frequently prescribed medications in pediatrics and adolescents (<20 years) and in adults and the elderly (≥20 years) in 2002–2003 and 2012–2013. (DOCX)

## References

[1] Wise J. Polypharmacy: a necessary evil. BMJ. 2013;347:f7033 10.1136/bmj.f7033 24286985

[2] Mallet L, Spinewine A, Huang A. The challenge of managing drug interactions in elderly people. Lancet. 2007;370(9582):185–91. 10.1016/S0140-6736(07)61092-7 17630042

[3] Richardson K, Ananou A, Lafortune L, Brayne C, Matthews FE. Variation over time in the association between polypharmacy and mortality in the older population. Drugs & Aging. 2011;28(7):547–60. 21721599 10.2165/11592000-000000000-00000

[4] Herr M, Robine JM, Pinot J, Arvieu JJ, Ankri J. Polypharmacy and frailty: prevalence, relationship, and impact on mortality in a French sample of 2350 old people. Pharmacoepidemiology and Drug Safety. 2015;24(6):637–46. 10.1002/pds.3772 25858336

[5] Jyrkka J, Enlund H, Lavikainen P, Sulkava R, Hartikainen S. Association of polypharmacy with nutritional status, functional ability and cognitive capacity over a three-year period in an elderly population. Pharmacoepidemiology and Drug Safety. 2011;20(5):514–22. 10.1002/pds.2116 21308855

[6] Lai SW, Lin CH, Liao KF, Su LT, Sung FC, Lin CC. Association between polypharmacy and dementia in older people: a population-based case-control study in Taiwan. Geriatrics & Gerontology International. 2012;12(3):491–8. 22233227 10.1111/j.1447-0594.2011.00800.x

[7] Laflamme L, Monarrez-Espino J, Johnell K, Elling B, Moller J. Type, number or both? A population-based matched case-control study on the risk of fall injuries among older people and number of medications beyond fall-inducing drugs. PLoS One. 2015;10(3):e0123390 10.1371/journal.pone.0123390 25815483 PMC4376700

[8] Kojima T, Akishita M, Nakamura T, Nomura K, Ogawa S, Iijima K, et al Polypharmacy as a risk for fall occurrence in geriatric outpatients. Geriatrics & Gerontology International. 2012;12(3):425–30. 22212467 10.1111/j.1447-0594.2011.00783.x

[9] Niikawa H, Okamura T, Ito K, Ura C, Miyamae F, Sakuma N, et al Association between polypharmacy and cognitive impairment in an elderly Japanese population residing in an urban community. Geriatrics & Gerontology International. 2017;17(9):1286–93. 27628036 10.1111/ggi.12862

[10] Hernandez J, Vargas ML. Adverse drug events in ambulatory care. New England Journal of Medicine. 2003;349(3):303–5; author reply -5. 10.1056/NEJM200307173490319 12867616

[11] Odubanjo E, Bennett K, Feely J. Influence of socioeconomic status on the quality of prescribing in the elderly—a population based study. British Journal of Clinical Pharmacology. 2004;58(5):496–502. 10.1111/j.1365-2125.2004.02179.x 15521897 PMC1884614

[12] Nandi A, Glymour MM, Subramanian SV. Association among socioeconomic status, health behaviors, and all-cause mortality in the United States. Epidemiology. 2014;25(2):170–7. 10.1097/EDE.0000000000000038 24487200

[13] Kivimaki M, Virtanen M, Kawachi I, Nyberg ST, Alfredsson L, Batty GD, et al Long working hours, socioeconomic status, and the risk of incident type 2 diabetes: a meta-analysis of published and unpublished data from 222 120 individuals. Lancet Diabetes & Endocrinology. 2015;3(1):27–34. 25262544 10.1016/S2213-8587(14)70178-0 PMC4286814

[14] Rawshani A, Svensson AM, Rosengren A, Eliasson B, Gudbjornsdottir S. Impact of socioeconomic status on cardiovascular disease and mortality in 24,947 individuals with type 1 diabetes. Diabetes Care. 2015;38(8):1518–27. 10.2337/dc15-0145 25972573

[15] Barnett K, Mercer SW, Norbury M, Watt G, Wyke S, Guthrie B. Epidemiology of multimorbidity and implications for health care, research, and medical education: a cross-sectional study. Lancet. 2012;380(9836):37–43. 10.1016/S0140-6736(12)60240-2 22579043

[16] Fried TR, O’Leary J, Towle V, Goldstein MK, Trentalange M, Martin DK. Health outcomes associated with polypharmacy in community-dwelling older adults: a systematic review. Journal of the American Geriatrics Society. 2014;62(12):2261–72. 10.1111/jgs.13153 25516023 PMC4270076

[17] Hajjar ER, Cafiero AC, Hanlon JT. Polypharmacy in elderly patients. American Journal of Geriatric Pharmacotherapy. 2007;5(4):345–51. 10.1016/j.amjopharm.2007.12.002 18179993

[18] Slabaugh SL, Maio V, Templin M, Abouzaid S. Prevalence and risk of polypharmacy among the elderly in an outpatient setting: a retrospective cohort study in the Emilia-Romagna region, Italy. Drugs & Aging. 2010;27(12):1019–28. 21087071 10.2165/11584990-000000000-00000

[19] Yoon YK, Park GC, An H, Chun BC, Sohn JW, Kim MJ. Trends of antibiotic consumption in Korea according to national reimbursement data (2008–2012): A population-based epidemiologic study. Medicine. 2015;94(46):e2100 10.1097/MD.0000000000002100 26579825 PMC4652834

[20] Hersh AL, Shapiro DJ, Pavia AT, Shah SS. Antibiotic prescribing in ambulatory pediatrics in the United States. Pediatrics. 2011;128(6):1053–61. 10.1542/peds.2011-1337 22065263

[21] Charlesworth CJ, Smit E, Lee DS, Alramadhan F, Odden MC. Polypharmacy among adults aged 65 years and older in the United States: 1988–2010. Journals of Gerontology Series A, Biological Sciences and Medical Sciences. 2015;70(8):989–95. 10.1093/gerona/glv013 PMC4573668 25733718

[22] Centre THaSCI. Prescriptions dispensed in the community, statistics for England, 2001–2011. Prescribing and Primary Care Services; 2012.

[23] Lee J, Lee JS, Park SH, Shin SA, Kim K. Cohort Profile: The National Health Insurance Service-National Sample Cohort (NHIS-NSC), South Korea. International Journal of Epidemiology. 2017;46(2):e15 10.1093/ije/dyv319 26822938

[24] Jones RS. "Health-Care Reform in Korea", OECD Economics Department Working Papers. Paris: OECD Publishing; 2010.

[25] Van den Bussche H, Koller D, Kolonko T, Hansen H, Wegscheider K, Glaeske G, et al Which chronic diseases and disease combinations are specific to multimorbidity in the elderly? Results of a claims data based cross-sectional study in Germany. BMC Public Health. 2011;11:101 10.1186/1471-2458-11-101 21320345 PMC3050745

[26] Jung KH OY LY, Sohn CK, Park BM, et al. Elderly Survey of Korea 2011. Seoul: Ministry of Health and Welfare. Korea Institute for Health and Social Affairs; 2012.

[27] Charlson ME, Pompei P, Ales KL, MacKenzie CR. A new method of classifying prognostic comorbidity in longitudinal studies: development and validation. Journal of Chronic Diseases. 1987;40(5):373–83. 3558716 10.1016/0021-9681(87)90171-8

[28] Kim DS, Bae G, Yoo SY, Kang M. Between government policy, clinical autonomy, and market demands: a qualitative study of the impact of the Prescribing Analysis System on behavior of physicians in South Korea. BMC Health Services Research. 2015;15:397 10.1186/s12913-015-1059-x 26392282 PMC4578427

[29] Kantor ED, Rehm CD, Haas JS, Chan AT, Giovannucci EL. Trends in prescription drug use among adults in the United States from 1999–2012. JAMA. 2015;314(17):1818–31. 10.1001/jama.2015.13766 26529160 PMC4752169

[30] Guthrie B, Makubate B, Hernandez-Santiago V, Dreischulte T. The rising tide of polypharmacy and drug-drug interactions: population database analysis 1995–2010. BMC Medicine. 2015;13:74 10.1186/s12916-015-0322-7 25889849 PMC4417329

[31] Nishtala PS, Salahudeen MS. Temporal trends in polypharmacy and hyperpolypharmacy in older New Zealanders over a 9-year period: 2005–2013. Gerontology. 2015;61(3):195–202. 10.1159/000368191 25428287

[32] Park J, Han E, Lee SO, Kim DS. Antibiotic use in South Korea from 2007 to 2014: A health insurance database-generated time series analysis. PLoS One. 2017;12(5):e0177435 10.1371/journal.pone.0177435 28520761 PMC5435228

[33] Youngster I, Avorn J, Belleudi V, Cantarutti A, Diez-Domingo J, Kirchmayer U, et al Antibiotic use in children—A cross-national analysis of 6 countries. Journal of Pediatrics. 2017;182:239–44.e1. 10.1016/j.jpeds.2016.11.027 28012694

[34] Statistics NCfH. Health, United States, 2016: With Chartbook on Long-term Trends in Health Hyattsville, MD: National Center for Health Statistics 2017 [http://www.cdc.gov/nchs/hus/contents2016.htm#079.] 28910066

[35] Rotermann M, Sanmartin C, Hennessy D, Arthur M. Health Reports: Prescription medication use by Canadians aged 6 to 79. Statistics Canada Catalogue no. 82-003-X; 2014. 24941315

[36] Caldwell P. Child survival: physical vulnerability and resilience in adversity in the European past and the contemporary Third World. Social Science & Medicine (1982). 1996;43(5):609–19. 8870127 10.1016/0277-9536(96)00109-8

[37] Simon AK, Hollander GA, McMichael A. Evolution of the immune system in humans from infancy to old age. Proceedings Biological Sciences. 2015;282(1821):20143085 10.1098/rspb.2014.3085 26702035 PMC4707740

[38] Kearns GL, Abdel-Rahman SM, Alander SW, Blowey DL, Leeder JS, Kauffman RE. Developmental pharmacology—drug disposition, action, and therapy in infants and children. New England Journal of Medicine. 2003;349(12):1157–67. 10.1056/NEJMra035092 13679531

[39] De Cock RF, Piana C, Krekels EH, Danhof M, Allegaert K, Knibbe CA. The role of population PK-PD modelling in paediatric clinical research. European Journal of Clinical Pharmacology. 2011;67 Suppl 1:5–16. 20340012 10.1007/s00228-009-0782-9 PMC3082690

[40] Kwon S. Thirty years of national health insurance in South Korea: lessons for achieving universal health care coverage. Health Policy and Planning. 2009;24(1):63–71. 10.1093/heapol/czn037 19004861

[41] Bae B, Choi BR, Song I. The impact of change from copayment to coinsurance on medical care usage and expenditure in outpatient setting in older Koreans. International Journal of Health Planning and Management. 2018;33(1):235–45. 10.1002/hpm.2416 28370318

[42] Joo J-M, Kwon S-M. Difference in outpatient medical expenditure and physician practice patterns between medicaid and health insurance patients. Health Policy and Management. 2009;19(3):125–41.

[43] Decrease in the number of outpatient visits after the change from co-payment to co-insurance system. Ministry of Health and Welfare. 2009; [http://www.mohw.go.kr/react/al/sal0301vw.jsp?PAR_MENU_ID=04&MENU_ID=0403&CONT_SEQ=203550&page=1]

[44] Song YJ. The South Korean health care system. Japan Medical Association Journal. 2009;52(3):206–9.

[45] Kim HA, Shin JY, Kim MH, Park BJ. Prevalence and predictors of polypharmacy among Korean elderly. PLoS One. 2014;9(6):e98043 10.1371/journal.pone.0098043 24915073 PMC4051604

[46] National Health Insurance Program Contributions: National Health Insurance Service; [http://www.nhis.or.kr/static/html/wbd/g/a/wbdga0404.html.]

[47] Wauters M, Elseviers M, Vaes B, Degryse J, Dalleur O, Vander Stichele R, et al Polypharmacy in a Belgian cohort of community-dwelling oldest old (80+). Acta Clinica Belgica. 2016;71(3):158–66. 10.1080/17843286.2016.1148298 27105401

[48] Franchi C, Tettamanti M, Pasina L, Djignefa CD, Fortino I, Bortolotti A, et al Changes in drug prescribing to Italian community-dwelling elderly people: the EPIFARM-Elderly Project 2000–2010. European Journal of Clinical Pharmacology. 2014;70(4):437–43. 10.1007/s00228-013-1621-6 24398968

[49] Bushardt RL, Massey EB, Simpson TW, Ariail JC, Simpson KN. Polypharmacy: misleading, but manageable. Clinical Interventions in Aging. 2008;3(2):383–9. 18686760 10.2147/cia.s2468 PMC2546482

[50] Mohammed S, Arabi A, El-Menyar A, Abdulkarim S, AlJundi A, Alqahtani A, et al Impact of polypharmacy on adherence to evidence-based medication in patients who underwent percutaneous coronary intervention. Current Vascular Pharmacology. 2016;14(4):388–93. 26517700 10.2174/1570161113666151030105805

[51] Onder G, Liperoti R, Foebel A, Fialova D, Topinkova E, van der Roest HG, et al Polypharmacy and mortality among nursing home residents with advanced cognitive impairment: results from the SHELTER study. Journal of the American Medical Directors Association. 2013;14(6):450.e7–12. 10.1016/j.jamda.2013.03.014 23647778

[52] Veehof L, Stewart R, Haaijer-Ruskamp F, Jong BM. The development of polypharmacy. A longitudinal study. Family Practice. 2000;17(3):261–7. 10846147 10.1093/fampra/17.3.261

[53] Narayan SW, Nishtala PS. Associations of potentially inappropriate medicine use with fall-related hospitalisations and primary care visits in older New Zealanders: A population-level study using the updated 2012 Beers Criteria. Drugs—real world outcomes. 2015;2(2):137–41. 10.1007/s40801-015-0020-y 27747763 PMC4883188

[54] Chan DC, Hao YT, Wu SC. Characteristics of outpatient prescriptions for frail Taiwanese elders with long-term care needs. Pharmacoepidemiology and drug safety. 2009;18(4):327–34. 10.1002/pds.1712 19180586

[55] HIRA. Prescribing Analysis Program Report. Health Insurance Review & Assessment Service. South Korea; 2014.

[56] Sohn HS, Jang S, Han E, Lee EJ, Shin SA, Lee JY. Patient factors affecting frequent potential unnecessary injection use in outpatient care setting. Archives of Pharmacal Research. 2015;38(7):1389–96. 10.1007/s12272-014-0406-5 24838381

[57] HIRA. Manual for Evaluation Project on Appropriate Prescribing. Health Insurance Review & Assessment Service. South Korea; 2007.

[58] Masnoon N, Shakib S, Kalisch-Ellett L, Caughey GE. What is polypharmacy? A systematic review of definitions. BMC Geriatrics. 2017;17(1):230 10.1186/s12877-017-0621-2 29017448 PMC5635569

[59] Immonen S, Valvanne J, Pitkala KH. The prevalence of potential alcohol-drug interactions in older adults. Scandinavian Journal of Primary Health Care. 2013;31(2):73–8. 10.3109/02813432.2013.788272 23621352 PMC3656398

