# Supplementary material for: Trends in polypharmacy over 12 years and changes in its social gradients in South Korea
Source: PLoS One. 2018 Sep 18;13(9):e0204018. doi: 10.1371/journal.pone.0204018 (PMC6143262; doi:10.1371/journal.pone.0204018)
Supplement: S1 File — (DOCX) [file pone.0204018.s001.docx]

S1 File. The 51 chronic conditions included in the multimorbidity count

| **Name of condition** | **Mental/physical health condition** | **Variable definition (ICD-10)** |
| --- | --- | --- |
| Allergies | Physical | J30, K52.2, K90.0, T78.1, T78.4, T88.7 |
| Anemia | Physical | D50, D51, D52, D53, D55, D56, D57, D58, D59.0, D59.1, D59.2, D59.4, D59.5, D59.6, D59.7, D59.8, D59.9, D60.0, D60.8, D60.9, D61, D63, D64 |
| Anxiety | Mental | F40, F41 |
| Asthma/COPD | Physical | J44, J45, J47 |
| Atherosclerosis/PAOD | Physical | I65, I66, I67, I70, I73 |
| Cardiac arrhythmias | Physical | I44, I45, I46, I47, I48, I49 |
| Cardiac insufficiency | Physical | I50 |
| Cardiac valve disorders | Physical | I34, I35, I36, I37 |
| Cataract | Physical | H25, H26, H28, Q12 |
| Cerebral ischemia/Chronic stroke | Physical | I60, I61, I62, I63, I64, I69, G45 |
| Chronic bronchitis/Emphysema | Physical | J40, J41, J42, J43 |
| Chronic cholecystitis/Gallstones | Physical | K80, K81.1 |
| Chronic gastritis/GERD | Physical | K21, K25, K26, K27, K28, K29.2, K29.3, K29.4, K29.5, K29.6, K29.7, K29.8, K29.9 |
| Chronic ischemic heart disease | Physical | I20, I21, I25 |
| Chronic low back pain | Physical | M40, M41, M42, M43, M44, M45, M47, M48.0, M48.1, M48.2, M48.5, M48.6, M48.7, M48.8, M48.9, M50, M51, M52, M53, M54 |
| Chronic otitis media | Physical | H65.2, H65.3, H66.1, H66.2 |
| Dementia | Mental | F00, F01, F02, F03, F05.1, G30, G31, R54 |
| Depression | Mental | F32, F33 |
| Diabetes mellitus | Physical | E10, E11, E12, E13, E14 |
| Diseases of the skin and subcutaneous tissue | Physical | L00–L99 |
| Dizziness | Physical | H81, H82, R42 |
| Fracture/Dislocation/Sequela | Physical | S02, S12, S22, S32, S42, S52, S62, S72, S82, S92, T02, T08, T10, T12, T14.2, T14.3, X59, Y85, Y86, Y87, Y88, Y89 |
| Glaucoma | Physical | H40, H42 |
| Hemorrhoids | Physical | I84 |
| Hypertension | Physical | I10, I11, I12, I13, I14, I15 |
| Hypotension | Physical | I95 |
| Insomnia | Mental | G47, F51 |
| Intestinal diverticulosis | Physical | K57 |
| Lipid metabolism disorders | Physical | E78 |
| Liver disease | Physical | K70, K71.3–K71.5, K71.7, K72.1, K72.9, K73, K74, K76 |
| Lower limb varicosis | Physical | I83, I87.2 |
| Migraine/chronic headache | Physical | G43, G44 |
| Neuropathies | Physical | G50, G51, G52, G53, G54, G55, G56, G57, G58, G59, G60, G61, G62, G63, G64 |
| Non-inflammatory gynecological problems | Physical | N81, N84, N85, N86, N87, N88, N89, N90, N93, N95 |
| Obesity | Physical | E66 |
| Osteoarthrosis | Physical | M15, M16, M17, M18, M19 |
| Osteoporosis | Physical | M80, M81, M82 |
| Parkinson’s disease | Physical | G20, G21, G22 |
| Prostatic hyperplasia | Physical | N40 |
| Purine, pyrimidine metabolism disorders/gout | Physical | E79, M10 |
| Renal insufficiency | Physical | N18, N19 |
| Rheumatoid arthritis/chronic polyarthritis | Physical | M05, M06, M79 |
| Severe hearing loss | Physical | H90, H91.0, H91.1, H91.3, H91.8, H91.9 |
| Severe vision reduction | Physical | H17, H18, H27, H31, H33, H34.1, H34.2, H34.8, H34.9, H35, H36, H43, H47, H54 |
| Sexual dysfunction | Physical | F52, N48.4 |
| Somatoform disorders | Physical | F45 |
| Thyroid dysfunction | Physical | E01, E06, E07 |
| Tobacco abuse | Mental | F17 |
| Tuberculosis | Physical | A15, A16, A17, A18 |
| Urinary incontinence | Physical | N39.3, N39.4, R32 |
| Urinary tract calculi | Physical | N20 |
| * The definitions of multimorbidity were adopted from those used in a previous publication (Kim *et al.*, 2014) (https://doi.org/10.1371/journal.pone.0098043) | | |
