# Supplementary material for: Trends in polypharmacy over 12 years and changes in its social gradients in South Korea
Source: PLoS One. 2018 Sep 18;13(9):e0204018. doi: 10.1371/journal.pone.0204018 (PMC6143262; doi:10.1371/journal.pone.0204018)
Supplement: S2 File — (DOCX) [file pone.0204018.s002.docx]

S2 File. Changes in the prescription trends of polypharmacy and non-polypharmacy between 2002 and 2013.

|  | **Number of Prescriptions** | | |  |
| --- | --- | --- | --- | --- |
|  |  |  |  |  |
|  | **Total** | **Polypharmacy**† | **Non-polypharmacy** | **Mean (SD)** |
| **Year** |  |  |  |  |
| 2002 | 4,578,987 | 1,159,901 | 3,419,086 | 4.4 (2.0) |
| 2003 | 4,663,726 | 1,250,810 | 3,412,916 | 4.5 (2.3) |
| 2004 | 5,034,977 | 1,316,818 | 3,718,159 | 4.5 (2.3) |
| 2005 | 5,459,827 | 1,438,684 | 4,021,143 | 4.5 (2.3) |
| 2006 | 5,667,715 | 1,450,058 | 4,217,657 | 4.4 (2.3) |
| 2007 | 7,275,144 | 1,474,237 | 5,800,907 | 4.2 (2.0) |
| 2008 | 8,318,737 | 1,434,576 | 6,884,161 | 4.0 (1.8) |
| 2009 | 8,711,904 | 1,420,458 | 7,291,446 | 4.0 (1.8) |
| 2010 | 8,913,995 | 1,451,677 | 7,462,318 | 4.0 (1.8) |
| 2011 | 9,534,656 | 1,467,368 | 8,067,288 | 3.9 (1.8) |
| 2012 | 10,036,698 | 1,439,518 | 8,597,180 | 3.8 (1.8) |
| 2013 | 9,981,730 | 1,430,723 | 8,551,007 | 3.8 (1.8) |

† Polypharmacy was defined as the concomitant prescription of ≥6 distinct medications
