# Supplementary material for: Trends in polypharmacy over 12 years and changes in its social gradients in South Korea
Source: PLoS One. 2018 Sep 18;13(9):e0204018. doi: 10.1371/journal.pone.0204018 (PMC6143262; doi:10.1371/journal.pone.0204018)
Supplement: S3 File — (DOCX) [file pone.0204018.s003.docx]

S3 File. Changes in the prevalence of polypharmacy, defined as ≥5 medications in pediatrics and adolescents (<20 years of age) and in adults and the elderly (≥20 years of age) between 2002 and 2013.

|  |  | **Pediatrics and adolescents**  **(<20 years)** | | | **Adults and elderly**  **(≥20 years)** | | |
| --- | --- | --- | --- | --- | --- | --- | --- |
|  | **Age-standardized prevalence^*^** | **Prevalence**^†^ | **Total**  **(N)** | **Poly (N) ^‡^** | **Prevalence** | **Total**  **(N)** | **Poly (N)** |
| **Year** |  |  |  |  |  |  |  |
| 2002 | 80.57% | 85.15% | 236,399 | 201,300 | 71.96% | 533,648 | 383,987 |
| 2003 | 75.58% | 80.50% | 219,141 | 176,417 | 70.81% | 543,067 | 384,567 |
| 2004 | 78.99% | 82.09% | 219,939 | 180,541 | 72.46% | 565,998 | 410,132 |
| 2005 | 82.72% | 82.91% | 216,886 | 179,830 | 74.60% | 588,092 | 438,699 |
| 2006 | 83.31% | 83.01% | 210,687 | 174,895 | 74.93% | 591,303 | 443,064 |
| 2007 | 80.10% | 79.64% | 208,884 | 166,351 | 74.07% | 614,901 | 455,447 |
| 2008 | 76.52% | 78.06% | 204,662 | 159,767 | 72.43% | 624,777 | 452,540 |
| 2009 | 77.30% | 80.38% | 208,518 | 167,615 | 72.16% | 639,253 | 461,282 |
| 2010 | 77.83% | 80.60% | 199,900 | 161,116 | 72.44% | 635,737 | 460,514 |
| 2011 | 73.37% | 76.46% | 200,928 | 153,621 | 71.02% | 667,742 | 474,255 |
| 2012 | 72.37% | 75.88% | 197,825 | 150,104 | 70.66% | 680,235 | 480,682 |
| 2013 | 71.44% | 75.72% | 192,172 | 145,508 | 70.24% | 687,607 | 483,000 |

Abbreviations: Poly, polypharmacy

^*^ Yearly prevalence was standardized to the age distributions in 2013.

^†^ Prevalence was by dividing the sum of polypharmacy patients by the total number of outpatients in the respective year.

**^‡^** Polypharmacy was defined as the concomitant prescription of ≥5 distinct medications on a single prescription without a given duration of time.
