# Supplementary material for: Trends in polypharmacy over 12 years and changes in its social gradients in South Korea
Source: PLoS One. 2018 Sep 18;13(9):e0204018. doi: 10.1371/journal.pone.0204018 (PMC6143262; doi:10.1371/journal.pone.0204018)
Supplement: S4 File — (DOCX) [file pone.0204018.s004.docx]

S4 File. Changes in the prevalence of polypharmacy in non-cancer patients between 2002 and 2013.

|  |  | **Pediatrics and adolescents (<20 years)** | | |  | **Adults and elderly (≥20 years)** | | | | |
| --- | --- | --- | --- | --- | --- | --- | --- | --- | --- | --- |
|  |  |  |  |  |  |  |  | | |  |
|  | **Age-standardized prevalence*** | **Prevalence**† | **Total**  **(N)** | **Polypharmacy‡**  **(N)** |  | **Prevalence** | | **Total**  **(N)** | **Polypharmacy**  **(N)** | |
| **Year** |  |  |  |  |  |  | |  |  | |
| 2002 | 66.1% | 65.3% | 235,651 | 153,806 |  | 65.3% | | 497,684 | 239,104 | |
| 2003 | 66.0% | 62.7% | 218,494 | 136,982 |  | 65.3% | | 506,652 | 251,379 | |
| 2004 | 69.5% | 64.0% | 219,354 | 140,327 |  | 65.3% | | 529,553 | 270,066 | |
| 2005 | 74.9% | 64.9% | 216,363 | 140,396 |  | 65.3% | | 551,796 | 295,731 | |
| 2006 | 74.6% | 64.4% | 210,217 | 135,443 |  | 65.3% | | 556,012 | 297,676 | |
| 2007 | 62.0% | 56.2% | 208,433 | 117,172 |  | 65.3% | | 579,784 | 288,361 | |
| 2008 | 50.3% | 50.2% | 204,222 | 102,589 |  | 65.3% | | 590,040 | 266,171 | |
| 2009 | 49.9% | 52.2% | 208,104 | 108,578 |  | 65.3% | | 605,395 | 267,328 | |
| 2010 | 51.2% | 53.0% | 199,515 | 105,718 |  | 65.3% | | 603,427 | 269,645 | |
| 2011 | 46.4% | 48.4% | 200,563 | 97,034 |  | 65.3% | | 634,964 | 276,116 | |
| 2012 | 43.9% | 46.8% | 197,483 | 92,423 |  | 65.3% | | 648,555 | 275,842 | |
| 2013 | 43.4% | 46.7% | 191,879 | 89,512 |  | 65.3% | | 657,234 | 279,218 | |

Non-cancer patients were defined as those who had no diagnosis records of cancer (ICD-10:C00–C97) in the outpatient setting within the indicated year.

* Yearly prevalence was standardized to the age distributions in 2013.

† Prevalence was calculated as the sum of polypharmacy patients as a numerator, and the total number of outpatients as a denominator

**‡** Polypharmacy was defined as the concomitant prescription of ≥6 distinct medications on a single prescription without a given duration of time
