# Supplementary material for: Trends in polypharmacy over 12 years and changes in its social gradients in South Korea
Source: PLoS One. 2018 Sep 18;13(9):e0204018. doi: 10.1371/journal.pone.0204018 (PMC6143262; doi:10.1371/journal.pone.0204018)
Supplement: S5 File — (DOCX) [file pone.0204018.s005.docx]

S5 File. Sensitivity analysis of the association between participant characteristics, including insurance type, and polypharmacy (≥6 medications) in 2002–2003 and 2012–2013.

|  | **2002–2003**  **(N=880,781)** | | **2012–2013**  **(N=953,648)** | |
| --- | --- | --- | --- | --- |
|  | **Pediatrics and adolescents (<20)**  **N=258,559**  **aOR (95% CI)** | **Adults and**  **elderly (≥20)**  **N=622,222**  **aOR (95% CI)** | **Pediatrics and adolescents (<20)**  **N=206,668**  **aOR (95% CI)** | **Adults and**  **elderly (≥20)**  **N=746,980**  **aOR (95% CI)** |
| **Male (reference)** |  |  |  |  |
| Female | 1.08 (1.06–1.10) | 1.38 (1.37–1.40) | 1.03 (1.01–1.05) | 1.24 (1.23–1.26) |
| **Insurance type**^†^ |  |  |  |  |
| **NHI beneficiary (reference)** |  |  |  |  |
| Medical aid | 0.49 (0.43–0.56) | 0.61 (0.55–0.67) | 0.91 (0.87–0.97) | 1.41 (1.36–1.46) |
| **Multimorbidity** |  |  |  |  |
| **0 (reference)** |  |  |  |  |
| 1 | 2.05 (2.01–2.09) | 1.77 (1.74–1.80) | 2.40 (2.30–2.49) | 1.87 (1.82–1.92) |
| 2–4 | 7.48 (7.30–7.62) | 5.23 (5.15–5.32) | 8.01 (7.73–8.31) | 5.32 (5.19–5.46) |
| 5–7 | 23.66 (19.39–28.89) | 20.11 (19.56–20.67) | 15.88 (14.90–16.93) | 14.69 (14.30–15.09) |
| ≥8 | 6.85 (2.01–22.80) | 67.91 (62.82–73.42) | 26.58 (16.84–41.97) | 44.11 (42.68–45.59) |

Abbreviations: aOR, adjusted odds ratio; CI, confidence interval; NHI, National Health Insurance

^†^ Insurance type was classified by patients’ beneficiary status in the NHI: 0th decile = medical aid recipient and 1st decile-10th decile = National Health Insurance beneficiary.
