# Supplementary material for: Trends in polypharmacy over 12 years and changes in its social gradients in South Korea
Source: PLoS One. 2018 Sep 18;13(9):e0204018. doi: 10.1371/journal.pone.0204018 (PMC6143262; doi:10.1371/journal.pone.0204018)
Supplement: S6 File — (DOCX) [file pone.0204018.s006.docx]

S6 File. The 20 most frequently prescribed medications in pediatrics and adolescents (<20 years), and in adults and the elderly (≥20 years) in 2002–2003 and 2012–2013.

| 2002–2003 | | | | 2012–2013 | | | |
| --- | --- | --- | --- | --- | --- | --- | --- |
| Pediatrics  No. of prescriptions (%) | | Adults  No. of prescriptions (%) | | Pediatrics  No. of prescriptions (%) | | Adults  No. of prescriptions (%) | |
| Mean prescription days= 2.7(±3.5) | | Mean prescription days= 8.6(±13.5) | | Mean prescription days= 3.4(±4.8) | | Mean prescription days= 13.2(±20.8) | |
|  |  |  |  |  |  |  |  |
| Acetaminophen | 920,126 (6.3) | Acetaminophen | 1,013,334 (3.8) | Chlorpheniramine | 1,906,140 (9.7) | Streptodornase | 2,133,855 (3.8) |
| Amoxicillin | 731,374 (5.0) | Cimetidine | 785,231 (3.0) | Acetaminophen | 1,071,438 (5.4) | Acetaminophen | 1,849,401 (3.3) |
| Streptokinase Streptodornase | 674,741 (4.6) | Dl-Methylephedrine Hydrochloride | 705,950 (2.7) | Amoxicillin | 1,010,054 (5.1) | Chlorpheniramine | 1,656,391 (2.9) |
| Ambroxol Hcl | 606,679 (4.2) | Streptodornase | 613,846 (2.3) | Pseudoephedrine | 982,589 (5.0) | Mosapride | 1,397,246 (2.5) |
| Brompheniramine | 544,082 (3.7) | Amoxicillin | 549,153 (2.1) | Streptokinase Streptodornase | 964,507 (4.9) | Loxoprofen | 1,383,889 (2.5) |
| Chlorpheniramine | 542,014 (3.7) | Serratiopeptidase | 514,870 (1.9) | Acetylcysteine | 867,568 (4.4) | Rebamipide | 1,339,491 (2.4) |
| Pseudoephedrine | 527,526 (3.6) | Aluminium Hydroxide Gel | 477,314 (1.8) | Ambroxol Hcl | 546,554 (2.8) | Cimetidine | 1,248,793 (2.2) |
| Dl-Methylephedrine Hydrochloride | 476,624 (3.3) | Diazepam | 450,087 (1.7) | Levocetirizine | 481,307 (2.4) | Ranitidine | 1,187,595 (2.1) |
| Cefaclor | 445,367 (3.0) | Almagate | 423,362 (1.6) | Formoterol Fumarate | 442,055 (2.2) | Almagate | 1,009,599 (1.8) |
| Ibuprofen | 425,997 (2.9) | Acetylcysteine | 386,897 (1.5) | Levodropropizine | 421,746 (2.1) | Acetylcysteine | 972,585 (1.7) |
| Acetylcysteine | 347,524 (2.4) | Prednisolone | 366,298 (1.4) | Dried Ivy Leaf Ext | 399,686 (2.0) | Pseudoephedrine | 916,483 (1.6) |
| Formoterol Fumarate | 324,603 (2.2) | Domperidone | 362,588 (1.4) | Dexibuprofen | 368,812 (1.9) | Aceclofenac | 904,066 (1.6) |
| Lactobacillus Fermentum | 558,300 (3.8) | Talniflumate | 352,861 (1.3) | Lactobacillus Fermentum | 368,475 (1.9) | Cefaclor | 810,529 (1.4) |
| L-Carbocysteine | 209,882 (1.4) | Levosulpiride | 329,843 (1.2) | Aminoacetic Acid | 360,309 (1.8) | Talniflumate | 778,590 (1.4) |
| Mequitazine | 184,659 (1.3) | Ofloxacin | 310,890 (1.2) | Cefaclor | 356,754 (1.8) | Levosulpiride | 775,638 (1.4) |
| Serratiopeptidase | 170,891 (1.2) | Brompheniramine | 301,242 (1.1) | Ibuprofen | 326,600 (1.7) | Dexibuprofen | 770,803 (1.4) |
| Hederae Helicis Folia | 148,267 (1.0) | Loxoprofen | 300,793 (1.1) | Montelukast | 298,642 (1.5) | Aspirin | 764,636 (1.4) |
| Trimebutine Maleate | 147,944 (1.0) | Biodiastase | 291,152 (1.1) | Pelargonium Sidoides Ext | 296,789 (1.5) | Eperisone | 656,106 (1.2) |
| Prednisolone | 147,560 (1.0) | Cefaclor | 286,134 (1.1) | Mequitazine | 274,920 (1.4) | Methylprednisolone | 600,556 (1.1) |
| Piprinhydrinate | 137,198 (0.9) | Mefenamic Acid | 259,512 (1.0) | Clarithromycin | 268,948 (1.4) | Levocetirizine | 595,352 (1.1) |
